# Pronostic visuel et évolution réfractive après chirurgie de la cataracte congénitale avec implantation primaire: étude d'une série de 108 cas

**DOI:** 10.11604/pamj.2013.16.51.2713

**Published:** 2013-10-13

**Authors:** Zouheir Hafidi, Wafaa Ibrahimy, Samir Ahid, Hanan Handor, Lalla Ouafae Cherkaoui, Zahid Bencherif, Mina Laghmari, Btissam Ouazzanni, Noureddine Boutimzine, Rajae Daoudi

**Affiliations:** 1 université Mohammed V Souissi, service d'ophtalmologie A de l'hôpital des spécialités, Centre hospitalier universitaire, Rabat, Maroc; 2 Université Mohammed V Souissi, laboratoire de biostatistiques, faculté de médecine, Rabat, Maroc

**Keywords:** Cataracte congénitale, bilatérale, unilatérale, réfraction, acuité visuelle, congenital cataract, bilateral, unilateral, refraction, visual acuity

## Abstract

La cataracte congénitale constitue la cause la plus fréquente de cécité évitable chez les enfants. Le but de cette étude est d’évaluer le pronostic réfractif et fonctionnel, des enfants opérés de cataracte congénitale avec implantation. Il s'agit d'une étude rétrospective de 108 enfants, dont 85 cataractes bilatérales, 23 unilatérales opérés entre 2007 et 2011. La réfraction a été mesurée à 1 mois, 3 mois, 6 mois, 1 an, 2 ans, 3 ans et/ou 4 ans en post-opératoire. La meilleure acuité visuelle corrigée, ainsi que l'incidence des complications post-opératoires ont été analysé. L’âge moyen de la chirurgie était de 25 mois avec une durée moyenne de suivi de 3,17 ans. Les complications retrouvées étaient l'inflammation, la prolifération secondaire, et le glaucome. L'acuité visuelle (AV) moyenne corrigée finale était de 5,75/10e pour les formes bilatérales, et de 4,16/10e pour les unilatérales (p = 0,001). Les facteurs de mauvais pronostic retrouvés étaient l’âge tardif de la chirurgie, la densité de la cataracte et la survenue de complications (p = 0,001). L'incidence des complications post-opératoires était significativement plus élevé chez les enfants opérés à un jeune âge (p = 0,001). Les facteurs de mauvais pronostic visuel chez les enfants opérés pour cataracte congénitale avec implantation, sont représentés par le caractère unilatéral de la cataracte, l’âge tardif de la chirurgie, la densité de la cataracte et la survenue de complications post opératoires.

## Introduction

La cataracte congénitale est l'une des principales causes de cécité évitable chez l'enfant [1]. On estime à 200.000 le nombre d'enfants à travers le monde au stade de cécité légale en rapport avec la cataracte. De ce fait sa prise en charge constitue la priorité du programme VISION 2020, initiative de l'OMS à l’échelon mondial pour l’élimination des principales causes de cécité évitable [2]. En effet, la gestion de cette pathologie continue de défier les ophtalmopédiatres, malgré les progrès enregistrés dans son approche diagnostique et thérapeutique [3]; car prévoir la croissance axiale d'un œil opéré et les changements réfractifs qui l'accompagnent constitue un véritable obstacle [4]. Une prise en charge chirurgicale précoce associée à une rééducation vigoureuse de l'amblyopie constituent les principaux piliers d'un bon pronostic fonctionnel, mais en raison du retard diagnostique, et de la faible compliance des parents au suivi post opératoire, le pronostic visuel des enfants atteints de cataractes congénitales reste défavorable. Le but de la présente étude est d’évaluer le pronostic visuel, et les facteurs qui les déterminent, ainsi que l’évolution réfractive chez des patients opérés pour cataracte congénitale.

## Méthodes

Il s'agit d'une étude rétrospective de 108 dossiers d'enfants opérés pour cataracte congénitale, dont 85 cataractes bilatérales et 23 cataractes unilatérales (193 yeux au total) colligées entre janvier 2007 et Décembre 2011. Les critères d'inclusion étaient: 1) Tous les enfants opérés de cataracte congénitale avec implantation primaire quel que soit l’âge de découverte et de prise en charge 2) Un suivi d'au moins 2 années. Tous les patients ont bénéficié d'un examen clinique: précisant le type anatomique de la cataracte, et l'existence d'une anomalies oculaires associées, l'examen du FO est réalisé si l’état des milieux le permet, dans la situation inverse une échographie en mode B est effectuée à but diagnostique. Cet examen est complété, souvent sous sédation, d'une biométrie (échographie A) et d'une kératométrie (auto-réfractomètre portable Nikon Rétinomax^®^), permettant, selon la formule SRK-II, le calcul de la puissance de l'implant emmétropisant théorique au moment de l'examen, La valeur obtenue est alors sous-corrigée selon l’âge pour obtenir une emmétropie à l’âge de 5 ans, selon le schéma de De Laage.

Le traitement chirurgical a consisté en une phaco-aspiration avec rhexis postérieur et vitréctomie antérieure, puis mise en place d'un implant pliable dans le sac. Un traitement anti-inflammatoire à base de corticothérapie topique horaire a été instauré chez tous les patients en postopératoire immédiat. Un examen sous sédation a été réalisé 1mois en post-opératoire pendant lequel on réalise: une ablation du fil, une réfraction objective et un examen ophtalmologique complet avec fond d’œil. Le traitement d'amblyopie est entrepris après l'ablation du fil, l'AV finale a pu être chiffré chez 100 patients grâce à l’échelle de Pigassou pour les enfants avec un âge lors du suivi compris entre 3 ans et 5 ans, puis par l’échelle des E de Snellen pour les plus âgés. Nous avons relevé l'acuité visuelle et la réfraction de chaque enfant œil par œil à 1 mois en post opératoire, puis chaque 3 à 6 mois.

### Recueil des données et Analyse statistique

Les valeurs d'acuité visuelle recueillies ont été converties en équivalent logMAR, pour permettre leur analyse statistique, et les résultats définitifs ont été convertis à nouveau en valeur décimale afin d’être interprétés plus facilement par les cliniciens. Les données ont été saisies et analysées sur le logiciel SPSS 13.0.

Les tests statistiques utilisés sont: le test de coefficient de corrélation (r), le test t de Student ou le test non paramétrique de Mann Whitney pour la comparaison de deux moyennes, le test du khi carré pour la comparaison des pourcentages. Le test de KruisKallWallis a été utilisé pour la comparaison de plus de deux variables qualitatives à distribution non Gaussienne. Les valeurs d'acuité visuelle recueillies ont été converties en équivalent logMAR, pour permettre leur analyse statistique. Le seuil de significativité a été fixé à 0,05. L'analyse univariée par la méthode de régression linéaire simple a été utilisée pour la recherche des facteurs prédictifs du LogMAR final. Seuls les facteurs avec un seuil de signification p < 0, 001 ont été introduits dans l'analyse multi variée.

Les limites de la présente étude, sont représentées par: la chirurgie a été réalisée par plusieurs chirurgiens; le faible pourcentage des cataractes unilatérales.

## Résultats

### Résultats descriptifs

**Figure 1:**
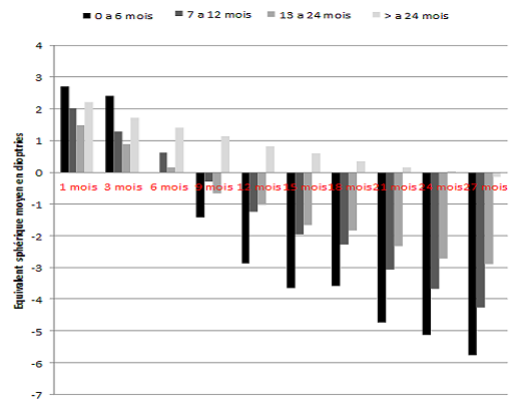
évolution de la réfraction post opératoire moyenne en fonction de l’âge au moment de la chirurgie

**Figure 2:**
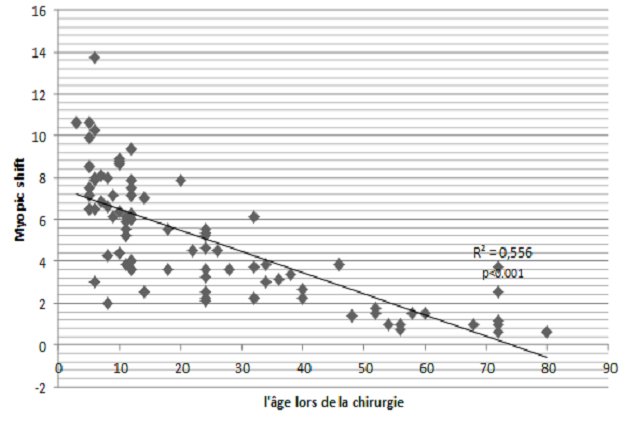
réprésentation de l'ensemble des résultats finaux du myopic shift en fonction de l’âge au moment de la chirurgie, démontrant des variations réfractives moins importantes à mesure que l'age au moment de la chirurgie augmente

108 enfants ont été opérés (193 yeux) dont 85 pour cataracte bilatérale (78,7%) et 23 pour cataracte unilatérale (21,3%). L’âge au moment de l'intervention variait entre 3 mois et 7 ans avec une médiane de 25 mois (24,60 mois pour les cataractes bilatérales et 26,30 mois pour les unilatérales). Une légère prédominance du sexe masculin a été notée, avec 62 garçons (57,7%) opérés, pour 46 filles (42,6%). La durée moyenne du suivi était de 38,06 mois (24 à 50 mois). Le strabisme et le nystagmus étaient respectivement présents chez 28 (25,9%) et 24 (22,2%) patients au moment du diagnostic. A la fin du suivi l'AV moyenne corrigée dans les cataractes bilatérales était de 0,21 (logMAR) pour le meilleur œil et de 0,27 (logMAR) pour l’æil adelphe, alors que pour les cataractes unilatérales elle était de 0,38 (logMAR). Les complications retrouvées étaient: l'inflammation dans 45 yeux (23,31%), la prolifération secondaire 26 yeux (13,47%), et le glaucome dans 9 yeux (4,6%). La moyenne de l’équivalent sphérique final, ainsi que l’évolution du myopic shift en fonction de l’âge ont montré une tendance à la myopisation avec des erreurs réfractives plus marquée chez les enfants opérés précocement (Figure 1, Figure 2).

### Analyse des résultats

**Figure 3:**
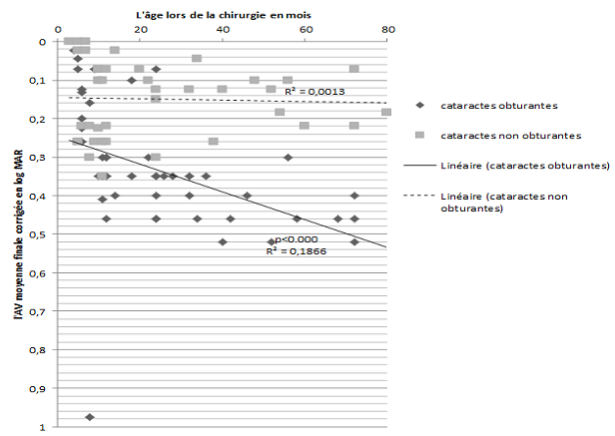
représentation de l'AV finale corrigée en fonction de l’âge au moment de la chirurgie et du type dela cataracte. L'AV finale corrigée (logMar) diminue significativement à mesure que l’âge au moment de la chirurgie augmente dans le groupe des cataracte obturantes

**Fonctionnels:** Nous avons noté une nette corrélation entre l'AV finale corrigée et la latéralité de la cataracte, avec un meilleur pronostic visuel des cataractes bilatérales comparativement à celui des cataractes unilatérales (p < 0,001). L'AV moyenne finale corrigée était significativement basse dans le groupe des patients ayant présentés des complications postopératoires comparativement au groupe des patients sans complications (p < 0,001). La relation entre l’âge au moment de l'intervention et l'acuité visuelle moyenne finale était fortement significative lors des cataractes obturantes (Figure 3). Dans une analyse multi-variée, les facteurs de mauvais pronostic retrouvés étaient l’âge tardif de la chirurgie la densité de la cataracte et la survenue de complications (Tableau 1).

**Tableau 1:** résultats de la régression multivariée incluant l’âge, la forme anatomique de la cataracte, la latéralité de la cataracte (uni ou bilatérale) et la survenue de complications

	Coefficient non standardisé	Signification	Intervalle de confiance à 95%	
	B		Borne inférieure	Borne supérieure
Age	2,781 E-03	0,000	0,001	0,004
Forme anatomique	-4,09 E-02	0,001	-0,065	-0,017
Latéralité de la cataracte	-0,171	0,000	-0,262	-0,080
complications	7,768 E-03	0,014	0,002	0,014

**Réfractifs:** La moyenne de l’équivalent sphérique final, ainsi que l’évolution du myopic shift en fonction de l’âge ont montré une tendance à la myopisation avec des erreurs réfractives plus marquées chez les enfants opérés précocement. Après analyse statistique on a noté une forte corrélation entre l’équivalent sphérique final et l’âge lors de la chirurgie, avec une erreur réfractive plus marquée lors d'une chirurgie précoce.

## Discussion

La cataracte congénitale est considérée comme la première cause de cécité évitable chez l'enfant [1]. Sa prise en charge constitue une priorité du programme VISION 2020: Le droit à la vue, l'initiative de l'OMS pour la lutte contre les principales causes de cécité évitable [2]. Toutefois, La gestion des cataractes congénitales pose encore des problèmes, et plusieurs questions demeurent un sujet de débat: Quand faut-il opérer?; Quelle technique chirurgicale faut-il adopter pour une meilleure évolution?; Quel est le meilleur moyen pour corriger l'aphakie? Faut-il implanter ou pas?; Quelle est la meilleure façon de déterminer la puissance de l'implant chez des enfants ou le globe est en pleine croissance, pour permettre une émmetropisation à l’âge adulte?

### Quand faut-il operer?

L'une des caractéristiques de la fonction visuelle est la diversité des éléments qui la constitue, ce qui rend, par conséquent, difficile la détermination d'une seule période sensible. [5] Toutefois il est bien établi qu'une anomalie de stimulation visuelle au cours des 3 premiers mois de vie, entraine une réduction des capacités fusionnelles [6]. On parle ainsi de période de latence, durant laquelle des perturbations de la stimulation visuelle, peuvent être à l'origine d'une amblyopie profonde [7].

Plusieurs études s'accordent alors sur l'intérêt d'une prise en charge précoce seul garant d'un pronostic fonctionnel favorable [8, 9]. En effet la période de latence varie en fonction de la densité et du caractère uni ou bilatéral de la cataracte, cette période semble être plus étroite en cas de cataracte unilatérale rendant la prise en charge plus urgente. Ceci est dû au fait que les patients présentant une cataracte unilatérale ont 2 facteurs prédisposant au développement de l′amblyopie: la rivalité binoculaire et la privation visuelle [10].

En cas de cataractes non obturantes, et comme il est difficile de mesurer avec précision l′acuité visuelle chez les enfants de bas âge, l′extraction de la cataracte doit être indiquée dans tous les cas avec une opacité d'au moins 3 mm sur l′axe visuel [11, 12].

Dans notre série on a remarqué une forte corrélation entre l’âge au moment de la chirurgie et l'AV moyenne corrigée finale, aussi bien au cours des cataractes bilatérales, que les cataractes unilatérales. L'AV finale se dégrade considérablement dans le groupe des cataractes obturantes à mesure que l’âge au moment de la chirurgie augmente. Ces résultats, similaires à ceux trouvés dans des études précédentes [8, 9] suggèrent que l’âge de l'enfant au moment de la chirurgie est le facteur le plus déterminant du pronostic visuel, avec un meilleur résultat fonctionnel pour les enfants opérés précocement.

L’âge moyen de nos patients (25 mois) au moment de la chirurgie est élevé comparativement à la majorité des séries publiées, ceci est dû à un retard de consultation par les parents, mais aussi et surtout à une absence de dépistage systématique à la naissance.

### Quelle technique chirurgicale faut-il adopter?

La technique de référence actuellement est la phacoémulsification avec capsulorhexis postérieur et vitrectomie antérieure [13]. Le capsulorhexis postérieur est pratiqué afin de prévenir L'opacification de la capsule postérieure, [14] qu'est l'une des complications les plus fréquente dans les suites post-opératoires de la cataracte congénitale, [15] grevant le pronostic fonctionnel [16].

Dans notre série tous les patients ont bénéficié d'un rhexis postérieur réalisé selon les habitudes de chaque chirurgien, soit complètement au kistytome soit ébauché par le kistytome et complété par une pince à rhexis ou bien réalisé par la sonde de vitréctomie (dans la majorité des cas), avec des résultats anatomiques comparables à moyen terme. 9,84% des yeux opérés ont présenté tout de même une cataracte secondaire ayant nécessité une reprise chirurgicale pour dégager l'axe visuel. Nous avons remarqué que le rhexis postérieur a tendance à s’élargir, probablement en rapport avec la nature élastique de la caplsule cristallinienne chez l'enfant, nous suggérons alors de réaliser un rhexis au vitréotome après avoir mis l'implant dans le sac, ce qui permet de garder le bénéfice de l'implantation.

### Quelle est le meilleur moyen pour corriger l'aphakie? Faut-il implanter ou pas?

L'implantation primaire en chambre postérieure est de plus en plus utilisée dans la correction de l'aphaquie chez l'enfant tout âge confondu [17].En plus de la réhabilitation rapide qu'elle permet sur le plan fonctionnel chez l'enfant, notamment dans les cataractes congénitales, [18, 19] l'implantation primaire offre plusieurs autres avantages, évoqués par Hiles [20] depuis les années 80, et qui comportent: l'amélioration de l'acuité visuelle finale par rapport aux autres modes de correction de l'aphaquie [21]; la réduction de l'anisométropie et de l'aniséiconie dans l'aphaquie unilatérale, la facilitation de la rééducation de l'amblyopie; la diminution de l'incidence du strabisme; la diminution des mouvements nystagmiformes; l'amélioration de la tolérance à la lumière; la suppression des inconvénients des lunettes et des lentilles de contact (pertes, aniséiconie, risque infectieux). De surcroit l'implantation permet une diminution de l'incidence des glaucomes des yeux implantés [22].

### Comment prevenir et gérer les complications post-opératoires?

Dans notre série les principales complications notées étaient: l'inflammation (20,20%); la prolifération secondaire (9,84%); l'hypertonie (3,63%).

**Les réactions inflammatoires:** comparée à une grande série américaine de 510 enfants, [17] notre série se caractérise par une plus grande incidence de l'inflammation, ceci peut être expliqué en grande partie par l'inobservance du traitement anti-inflammatoire par la majorité des patients. L'inflammation était significativement associée, dans notre étude, à un pronostic fonctionnel moins favorable, et constitue un facteur de risque de développement de cataracte secondaire. Il est important de rappeler que la gestion des réactions inflammatoires postopératoires passe par certaines mesures dont une bonne pratique chirurgicale en insistant sur: l'incision cornéenne pure exsangue, il y a donc moins de contact entre les facteurs d'inflammation apportés par le sang et la chambre antérieure; la diminution du traumatisme de l'iris est obtenue grâce à une bonne dilatation peropératoire [23], l'aspiration de toutes les masses cristalliniennes et le polissage de la capsule, -une vitrectomie antérieure systématique [24]; l'implantation dans le sac pour éviter tout contact uvéal source d'inflammation chronique; une cycloplégie et une corticothérapie locale permettent de juguler l'inflammation. Une corticothérapie générale de cinq à dix jours en post-opératoire semble réduire l'inflammation [23]. Certains auteurs utilisent des implants héparinés qui ont montrés leur efficacité dans la réduction de l'incidence des réactions inflammatoires chez l'enfant [25].

**Les hypertonies post opératoires:** Vu les progrès enregistrés dans la gestion de l'inflammation postopératoire, première cause des hypertonies post-opératoires, ces dernières sont du même coup devenu exceptionnelles [23, 26]. Parailleurs l'implantation primaire aurait un effet préventif contre l'hypertonie de l'enfant opéré de cataracte congénitale [22].

**Les cataractes secondaires:** Il est bien établi que la cataracte secondaire est inévitable en l'absence d'un capsulorhexis postérieur et d'une vitréctomie antérieure. [27] En effet les cellules résiduelles de la capsule peuvent migrer et proliférer sur la capsule et sur les faces postérieure et parfois antérieure de l'implant, pouvant obturer l'axe visuel et induire une amblyopie de déprivation irréversible. Ainsi Un capsulorhéxis postérieur et une vitrectomie antérieure doivent faire partie de toute chirurgie de la cataracte congénitale [24].

### Quelle est la meilleure façon de déterminer la puissance de l'implant chez des enfants ou le globe oculaire est en pleine expansion, pour permettre une émmetropisation à l’âge adulte?

Si l'implantation primaire est adopté par la plupart des auteurs, [28, 29] le choix de la puissance de l'implant intra-oculaire et d'une réfraction postopératoire cible porte encore à débat [30, 31]. La détermination de la puissance de l'implant passe par plusieurs étapes: le choix d'une réfraction postopératoire cible; le choix de la formule.

**Le choix d'une réfraction postopératoire cible:** en effet la croissance axiale du globe opéré et les changements réfractifs qui l'accompagnent sont difficiles à prévoir [4]. Normalement chez l'enfant le myopic shift varie peu ou pas au cours de la croissance oculaire pouvant aller de +0,4 dioptries (D) à la naissance à -0,5 D à l’âge adulte [32], malgré les grands changements de la longueur axiale allant de 16,8 mm à la naissance à 23,6 mm chez l′adulte [33]; ceci étant dû à une réduction de la puissance réfractive de l’æil pendant la croissance [34]. Notre attitude est de sous-corriger l’æil de façon à viser une emmétropie ou une légère myopie vers l’âge de cinq ans. Pour cela, la réfraction désirée était déterminée par une réduction de la puissance de l'implant selon le schéma de Delaage, généralement l'erreur réfractive était limitée, sauf pour le groupe d'enfants opérés avant l’âge de 1 ans chez qui l'erreur réfractive était beaucoup plus marquée. Ceci peut être expliqué par l'importance des variations en terme de longueur axiale qui s'opèrent au cours de la première année de vie [4, 32]; Sachant qu'au cours du développement du globe oculaire chez l'enfant, les changements de réfraction sont largement dus à la croissance axiale [35]. Toutefois une question reste un sujet de débat: dans quelle proportion l'implantation chez l'enfant changera-t-elle la croissance du globe? Les données de la littérature concernant ce sujet sont contradictoires, certains auteurs ont noté une diminution de la croissance axiale des yeux opérés, [36] tandis que d'autres ont rapporté une accélération de cette croissance avec une tendance à la myopisation; [37] d'autres encore n'ont pas retrouvé de différence dans la croissance axiale de l’æil pseudophaque et phaque. [38]

**Le choix de la formule:** Plusieurs formules sont utilisées pour calculer la puissance de l'implant nécessaire pour atteindre la réfraction souhaitée. Mais d'une part les formules utilisées actuellement chez l'enfant, proviennent essentiellement d′études chez l′adulte [35], d'autre part l’æil de l'enfant se caractérise par une longueur axiale réduite, une kératométrie élevée, avec un rapport profondeur de chambre antérieure / longueur axiale différent de celui de l′adulte. Alors qu'elle formule peut-ont utiliser pour optimiser le résultat réfractif? Là encore les données de la littérature ne fournissent pas de réponses claires, certaines études n'ont pas montré de différence entre les formules classiquement employées en terme de résultats réfractifs [31, 39], alors que d'autres se sont efforcées à prouver la supériorité de certaines formules [40]. Dans notre série nous avons utilisé la formule SRK II, elle nous a permis d'avoir des résultats réfractifs satisfaisants, avec un équivalent sphérique final ne dépassant pas les 6 dioptries tous âges confondus. Une des études les plus récentes [40] a démontré que le plus faible taux d′erreur réfractive a été obtenu avec la formule SRK II comparativement à d′autres formules incluant la SRK I, SRK T, Hoffer Q et la Holladay I. Enfin l′erreur réfractive chez l′enfant constiue un problème multifactoriel déterminé essentiellement par 4 facteurs [30]: erreurs lors de la mesure de la longueur axiale et de la kératométrie; erreurs chirurgicales, car l′implantation dans le sulcus résulte en un myopicshift supérieur à celui obtenu par une implantation dans le sac; erreurs dûes à la non standardisation de l′utilisation des formules de calcul de la puissance de l′implant chez l′enfant; erreurs réfractives induites par la croissance du globe oculaire chez l′enfant.

## Conclusion

Les résultats réfractifs de notre étude montrent que l'implantation primaire en chambre postérieure dans les cataractes congénitales chez les enfants, est réalisable et bénéfique à moyen terme. Par contre nos résultats fonctionnels sont relativement moins bons par rapport à d'autres études à cause de plusieurs facteurs: L’âge avancé de nos patients comparativement à d'autres séries. L'incidence plus élevée des complications post-opératoires notamment l'inflammation, due en grande partie à l'inobservance du traitement anti inflammatoire post-opératoire.
